# Influence of glass-based dental ceramic type and thickness with identical shade on the light transmittance and the degree of conversion of resin cement

**DOI:** 10.1038/s41368-017-0005-7

**Published:** 2018-03-02

**Authors:** Soram Oh, Su-Mi Shin, Hyun-Jung Kim, Janghyun Paek, Sung-Joon Kim, Tai Hyun Yoon, Sun-Young Kim

**Affiliations:** 1 Department of Conservative Dentistry, Graduate School, Kyung Hee University, Seoul, Korea; 2 Department of Prosthodontics, School of Dentistry, Kyung Hee University, Seoul, Korea; 3 Department of Dentistry, School of Medicine, Jeju National University, Jeju, Korea; 4 Department of Physics, Korea University, Seoul, Korea; 5 Department of Conservative Dentistry and Dental Research Institute, School of Dentistry, Seoul National University, Seoul, Korea

## Abstract

The purpose of this study was to assess the influence of the types and thicknesses of glass ceramic plates on light transmittance and compare the degrees of conversion (DC) of resin cement under the ceramic materials. Three ceramic plates with thicknesses of 0.5, 1.0, 2.0, and 4.0 mm were fabricated from each of five commercial ceramic blocks in shade A2: high-translucency and low-translucency IPS Empress CAD (Emp_HT and Emp_LT); high-translucency and low-translucency IPS e.max CAD (Emx_HT and Emx_LT); and Vita Mark II (Vita). The translucency parameter was obtained using a colorimeter. The light transmittance rate was measured using a photodetector attached to an optical power meter. The DC of a resin cement (Variolink N) underneath the ceramic plates was examined by Fourier transform infrared spectroscopy. The translucency parameter, light transmittance rate, and DC showed significant differences by ceramic type and thickness (*P* < 0.05). The Emp_HT specimens showed the highest light transmission and DCs, and the Emx_LT showed the least light transmission and the lowest DCs. The high-translucency Empress showed significantly higher DCs than the low-translucency types (*P* < 0.05), but there was no significant difference in e.max (*P* > 0.05). Both type and thickness of the glass ceramics significantly influenced the light transmittance and DC of the light-cured resin cement beneath the ceramic of the same shade.

## The cure is in the thickness

How well a dental filling or crown performs depends not only on the strength of ceramic material used but also on its thickness and type. Sun-Young Kim of Seoul National University and colleagues investigated how the thickness and type of glass-based ceramic affected how well it transmitted light and thus how well resin cement underneath it hardened. Orthodontists use a special ‘curing’ light to harden resin cement and create a strong bond between the ceramic material and the underlying mouth tissue. They shined light on different types of ceramic plates of similar shade but varying thickness and found that both their ability to transmit light and how well the resin cement underneath them hardened varied significantly. They conclude that orthodontists should not only choose ceramics for dental treatments based on their inherent strength, but should also consider how well they transmit light.

## Introduction

The use of dental ceramics is now increasing for restoring damaged, decayed, or missing teeth due to improvements in the physical properties of ceramics and advances in computer-aided design and computer-aided manufacturing (CAD/CAM) technology.^1–3^ Dental ceramics for all-ceramic restorations can be classified into two main categories by their compositions: glass-based and crystalline-based.^4^ Crystalline-based ceramics such as zirconia and alumina have stronger physical properties but poorer esthetic and bonding properties compared with glass-based ceramics.^5,6^ Glass-based ceramics show relatively better esthetics and bonding with resin cement, and therefore, they are mainly applied for reconstructing anterior teeth such as crowns and laminates and for intracoronal restorations in posterior teeth such as inlays and onlays.^4,7^ Feldspathic ceramic, a traditional glass-based ceramic, has been used for decades with good esthetic properties^7^. In the meantime, a leucite-reinforced glass ceramic, Empress® 1, was introduced in the late 1980s through many researchers’ and manufacturers’ efforts to develop a ceramic that was more resistant to crack propagation.^8^ A lithium disilicate-reinforced glass ceramic (IPS Empress® II, now called IPS e.max®), another enhanced glass-based ceramic, has now gained popularity as a material for restoring anterior and posterior teeth from laminates, inlays, and onlays to single crowns due to its superior physical properties.^9,10^ Monolithic ceramic blocks of all these glass-based ceramics are now available as CAD/CAM restorative materials for clinicians to choose based on their desired restorations and mechanical and optical properties.

Although lithium disilicate ceramic, e.max, has better mechanical strength than leucite-reinforced ceramic, Empress, and thus clinicians tend to prefer e.max to Empress,^4^ a ceramic with higher mechanical strength might not necessarily ensure better clinical performance. The clinical performance of ceramic restorations depends on adequate cementation to dental hard tissue with resin cement as well as the good mechanical properties of ceramic materials.^3,11^ The polymerization of resin cement under ceramic restorations is one of the most important factors in obtaining its optimal physical properties. Inadequate polymerization can yield poorer physical properties and faster degradation of cement finish line by acid, ultimately causing the de-bonding of restorations.^12,13^ Many previous studies reported that sufficient light curing is essential for achieving high polymerization even in dual-cured resin cements.^12–16^

The amount of light transmitted through ceramic restorations depends on the light intensity of the light curing unit and the thickness, type, and translucency of the ceramic materials.^17,18^ Although a number of studies have shown that increased thickness and darker shades of ceramic materials act as optical barriers to light reaching the cement,^19–21^ the level of light transmittance through dental ceramics and the consequent effects on the polymerization of resin cement have not yet been fully investigated. In particular, few studies have investigated the effects of different ceramic compositions (feldspathic, leucite-reinforced, and lithium disilicate-reinforced) and translucency (high and low) with identical shades on the light transmittance and degree of polymerization of resin cement.

Therefore, the objective of this study was to investigate the light transmittance through glass-based ceramics of identical shades with different compositions, translucency values, and thicknesses and to compare the degrees of polymerization of resin cement under those ceramic materials. The null hypothesis was that the light transmittance and degree of conversion (DC) of the resin cement through glass-based ceramics of identical shades would not be influenced by composition, translucency, or thickness.

## Results

### Translucency parameter

The translucency parameters for each ceramic specimen at the different thicknesses are shown in Table 1. The translucency parameter showed significant differences by ceramic plate type and thickness (*P* < 0.05), and the interactions between ceramic type and thickness were statistically significant (*P* < 0.05) (Table 2). Emp_HT showed the highest value of translucency parameter and Emx_LT showed the lowest in every thickness. Both the high-translucency types of Empress and e.max specimens showed higher translucency parameter values than the low-translucency types, and regardless of material, thicker specimens had lower translucency parameter values.

**Table 1:** Mean (SD) for translucency parameter and light transmittance rates (%) of different ceramic types and thicknesses

Dependent variable	Type	Thickness			
		0.5 mm	1.0 mm	2.0 mm	4.0 mm
Translucency parameter	Emp_HT^a^	26.06 (0.10)^A^	14.31 (0.06)^B^	6.28 (0.06)^C^	1.46 (0.16)^D^
	Emp_LT^d^	19.45 (0.05)^A^	11.30 (0.03)^B^	4.49 (0.06)^C^	0.94 (0.21)^D^
	Emx_HT^b^	20.79 (0.10)^A^	13.39 (0.04)^B^	5.27 (0.18)^C^	1.21 (0.20)^D^
	Emx_LT^e^	17.64 (0.05)^A^	9.74 (0.10)^B^	1.93 (0.03)^C^	0.58 (0.10)^D^
	Vita^c^	19.56 (0.06)^A^	11.98 (0.02)^B^	5.25 (0.16)^C^	1.33 (0.02)^D^
Light transmittance rates/%	Emp_HT^a^	28.23 (0.62)^A^	18.16 (0.17)^B^	9.75 (0.26)^C^	3.84 (0.04)^D^
	Emp_LT^c^	22.35 (0.13)^A^	13.72 (0.20)^B^	6.76 (0.04)^C^	1.97 (0.02)^D^
	Emx_HT^b^	23.44 (0.44)^A^	15.52 (0.07)^B^	8.94 (0.05)^C^	3.31 (0.01)^D^
	Emx_LT^d^	18.17 (0.48)^A^	11.42 (0.10)^B^	4.00 (0.46)^C^	1.10 (0.03)^D^
	Vita^b^	22.01 (0.15)^A^	15.59 (0.11)^B^	9.23 (0.16)^C^	3.47 (0.12)^D^

Different superscript (upper case for rows and lower case for columns) indicate statistical significance (*P* < 0.05) within each dependent variable

**Table 2:** Two-way ANOVA results of transmittance values, translucency parameter, and DC according to the ceramic type and thickness

Source	Sum of squares	Degrees of freedom	Mean squares	*F*	*P*-value
(a) Dependent variable: translucency parameter					
Ceramic type (A)	132.460	4	33.115	2912.072	0.000
Ceramic thickness (B)	3395.879	3	1131.960	99,542.097	0.000
A × B	62.629	12	5.219	458.955	0.000
Error	0.455	40	0.011	2912.072	
Total	9175.878	60			
(b) Dependent variable: transmittance value					
Ceramic type (A)	259.659	4	64.915	1028.327	0.000
Ceramic thickness (B)	3447.743	3	1149.248	18205.426	0.000
A × B	55.804	12	4.650	73.667	0.000
Error	2.525	40	0.063		
Total	12,476.918	60	64.915		
(c) Dependent variable: DC					
Ceramic type (A)	154.874	4	38.719	7.932	0.000
Ceramic thickness (B)	2208.586	3	736.195	150.823	0.000
A × B	140.101	12	11.675	2.392	0.013
Error	292.871	60	4.881		
Total	150,964.216	60			

### Light transmittance rates and light spectrum profiles through the ceramic plates

The light transmittance rates (%) for each ceramic specimen at the different thickness are also shown in Table 1. Transmitted light (%) showed significant differences by ceramic plate type and thickness (*P* < 0.05), and the interactions between ceramic type and thickness were also statistically significant (*P* < 0.05) (Table 2). In general, light transmittance was more effective in the high- rather than low-translucency ceramics. Emp_HT showed the highest transmittance rate and Emx_LT showed the lowest. Emp_LT transmitted significantly less light than Emx_HT or VITA. The light transmittance rate was less than 30% even at 0.5 mm thickness, and it decreased exponentially as the ceramic thickness increased. The light transmittance rate and translucency parameter had a very strong positive correlation (r = 0.993).

**Fig. 1:**
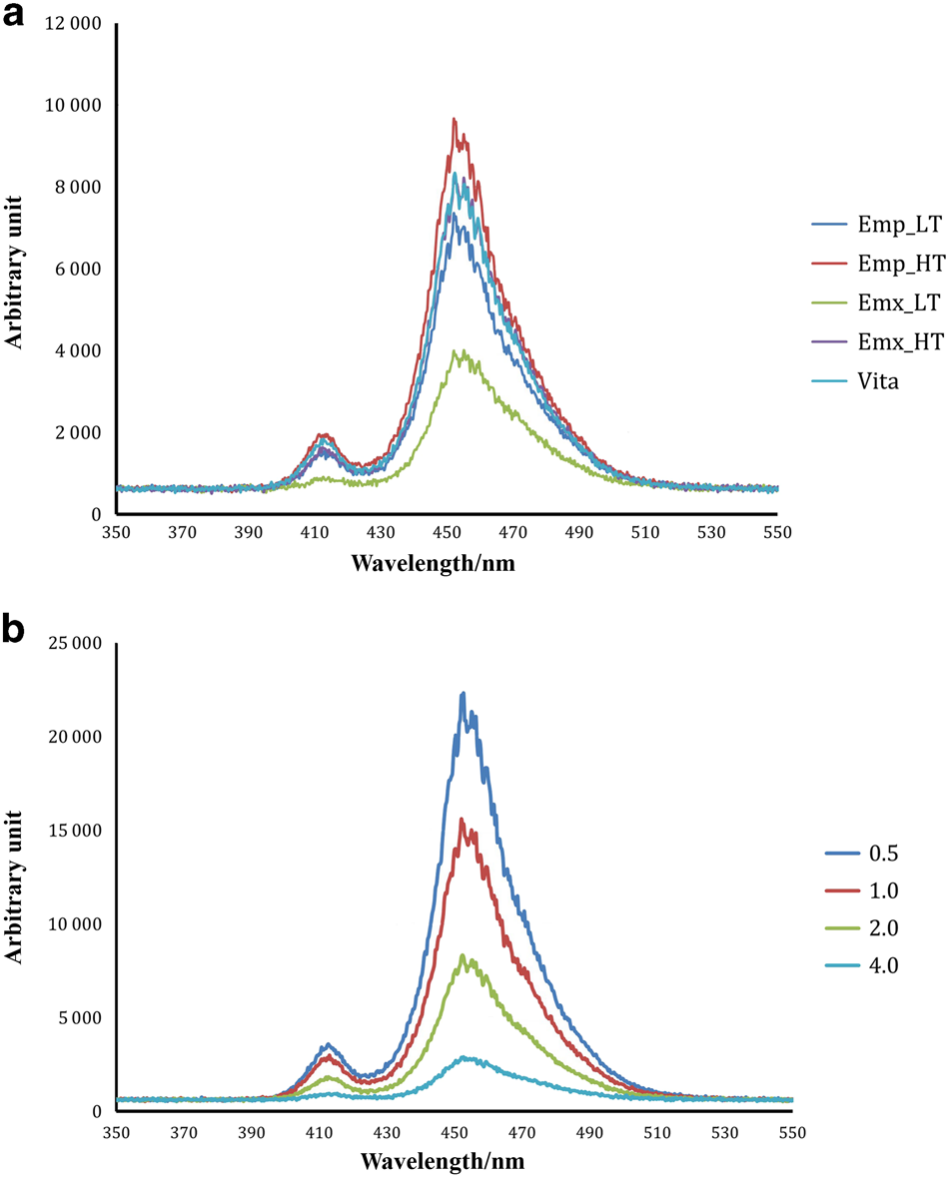
The representative light-spectrum profiles emitted. **a** Through the different ceramic types of 2.0 mm thickness and **b** through different thicknesses of Emp_LT

The representative light spectrum profiles emitted through the ceramic plates are depicted in Fig. 1. The peak spectrum was observed at 410 nm and 460 nm. Emx_LT showed the lowest peak value, and Emp_HT showed the highest (Fig. 1a), and as the ceramic thickness increased, the height of the peak decreased. When the Emp_LT was 4 mm thick, the peak spectrum at 410 nm was hardly observed (Fig. 1b).

### DC of resin cement

The DC of resin cement according to ceramic type and thickness are shown in Table 3. There were statistical differences by ceramic type and thickness (*P* < 0.05), and the interactions between the two were statistically significant (*P* < 0.05) (Table 2). Emp_HT showed the highest DC, and Emx_LT showed the lowest. Emp_HT showed no significant difference from Vita (*P* < 0.05) but yielded a higher DC than Emp_LT, Emx_HT, and Emx_LT (*P* < 0.05). Regarding the translucency type, the high-translucency types of Empress showed significantly higher DC than the low-translucency types (*P* < 0.05), but there were no significant differences between the two types in e.max (*P* < 0.05). Regardless of the material, DCs decreased significantly as the ceramic thickness increased (*P* < 0.05).

**Table 3:** Mean (SD) for degree of conversion (%) of light-cured resin cement under different ceramic types and thicknesses

Type	Thickness				
	0 mm	0.5 mm	1.0 mm	2.0 mm	4.0 mm
Emp_HT^a^	57.84 (3.71)	53.33 (2.63)^A^	48.91 (3.11)^B^	41.08 (3.46)^C^	37.73 (2.72)^D^
Emp_LT^b,c^		48.95 (3.40)^A^	47.08 (2.41)^B^	39.84 (4.76)^C^	35.23 (2.32)^D^
Emx_HT^b,c^		48.01 (0.97)^A^	44.08 (0.63)^B^	40.74 (0.83)^C^	36.90 (1.10)^D^
Emx_LT^c^		46.30 (1.50)^A^	43.70 (0.64)^B^	40.54 (1.29)^C^	35.03 (0.56)^D^
Vita^a,b^		51.59 (1.70)^A^	45.72 (1.46)^B^	42.47 (1.14)^C^	37.78 (0.98)^D^

Different superscript (upper case for rows and lower case for columns) indicate statistical significance (*P* < 0.05)

## Discussion

This study evaluated the influence of ceramic type and thickness on the light transmittance rates and assessed the DCs of the light-cured resin cements beneath the ceramics. A number of studies have investigated the effects of different shades and thicknesses on the light transmittance and the DCs of the underneath resin cement for the same ceramics;^16–18,22^ however, researchers have rarely investigated the effects of ceramic type in same shade on the light transmittance and the polymerization of resin cement. We speculated that the cements’ light transmittance and polymerization might differ according to the type of dental ceramic even in the same shade. Clinically, successful ceramic restorations require optimal physical properties of resin cement through adequate polymerization as well as the mechanical strength of the ceramic materials, which is the reason that light transmittance through ceramics should be considered. In the present study, we found that both ceramic type and thickness significantly influenced the light transmittance and the DC of the resin cement through the ceramic. Therefore, the null hypothesis of this study was rejected.

In this study, Light transmittance decreased exponentially with increasing ceramic plate thickness, which is consistent with previous studies.^17–19,22–24^ However, the light transmittance ratio was lower than it was in previous studies;^23,24^ in this study, the ratio was less than 30% even at 0.5 mm thickness and less than 5% at 4.0 mm (Fig. 1b). Kilinc et al.^23^ reported that 40%–50% of light was transmitted through 1 mm thick Empress ceramic in various shades, and Moraes et al.^24^ reported that approximately 42% of light was transmitted through 2 mm thick Empress shade A3. Zhang et al.^22^ reported that 37, 14, and 9% of irradiance was transmitted through 1, 2, and 3 mm thick e.max ceramics, respectively. In another study, 20% of the light was transmitted through 1.5 mm thick lithium disilicate ceramic of shade A2.^25^ Considering that in this study, the light guide tip and photodetector were separated by the corresponding ceramic thickness for measuring the original light emission for each depth (0.5, 1.0, 2.0, 4.0 mm), the light transmittance through the ceramics was notably low. If the original light intensity of the light curing unit had been set as the baseline value for all the thicknesses, the transmittance levels in this study would be much lower. Although we are not clear about why transmittance was lower in our study than in previous studies, the differences might have been related to the different shades of ceramic we used and different light curing units. Our having polished one surface of the ceramic plates but leaving the other surface rough could also have caused the different light attenuation conditions in this study. The decreasing light transmittance with increasing ceramic thickness was also reflected in the light spectrum results; the peak light spectrum profiles decreased considerably with increasing ceramic thickness (Fig. 1b). In particular, we rarely observed the 410 nm peak—one of the light curing unit’s two light emission peaks—in the spectrum through the 4.0 mm thick ceramics. The increasing ceramic thicknesses might raise concerns that sufficient polymerization will not be achieved by an additional co-initiator such as trimethylbenzoyl-diphenylphosphine oxide, which absorbs light near the 410 nm peak.^26^ As expected, the effect of light attenuation with increasing ceramic thickness was re-confirmed in the present study. Therefore, adequate polymerization of resin cement through thick ceramic plates might be difficult to achieve by light curing alone. Use of dual-cure resin cement is recommended in areas where light attenuation is anticipated to enhance polymerization by chemical curing.^17,23,27,28^

Ceramic type was also a determining factor for both light transmittance and light spectrum profiles. Emp_HT showed the highest light transmittance and light spectrum and Emx_LT showed the lowest for each ceramic thickness. There has been little research concerning the effects of ceramic type on transmitted light, and thus, it is difficult to compare the results of this study with those of previous studies. A plausible explanation for the differences in light transmittance by ceramic type is the different crystalline structures of ceramics including the compositions of filler and matrix.^29^ E.max is composed of 70% lithium disilicate by volume, which consists of many small, randomly oriented, interlocking plate-like crystals; light transmission through lithium disilicate ceramic is interrupted by the plate-like crystal.^4^ Vita Mark II is a feldspathic porcelain that contains less leucite crystal and more glass, and therefore light attenuation by a crystal component is relatively low.^4^ Given the fact that the ceramic type can cause a significant difference in light transmittance, this difference might have to be considered when choosing ceramic types, especially in restoring deep cavities in which the light transmittance is hindered.

Because highly translucent material is expected to have high light transmittance, we should have checked the linear relationship between the two. We obtained the light transmittance ratios by directly measuring the light irradiance, and we obtained the translucency parameters by measuring color against different backgrounds using a colorimeter.^30,31^ To our surprise, the light transmittance and translucency parameter had a nearly perfect positive linear correlation in spite of different measuring methods (Pearson’s correlation coefficient = 0.993), which has not been reported in previous studies to the best of our knowledge. Measuring light transmittance requires an expensive photo detecting machine, and it is a cumbersome procedure to obtain precise values, whereas the translucency parameter can be attained relatively quickly and easily with a simple colorimeter. Therefore, the translucency parameter appears to be an easier way of checking the light transmittance of restorative materials.

In the present study, the DC of resin cement differed significantly by ceramic type and thickness; the ceramic plates that transmitted more light led higher DCs of the underlying resin cement. Emp_HT presented the greatest DC of resin cement, and Emx_LT yielded the lowest DC, indicating the different light transmittance levels. A number of studies evaluated the degree of underlying resin cement polymerization at various ceramic thicknesses and reported conflicting results. Moraes et al.^24^ detected no significant differences in DC of dual-cured resin cements in 0.7, 1.4, and 2.0 mm thick ceramics, and Passos et al.^32^ also reported no significant differences in hardness between 1.0 and 3.0 mm thick ceramic. By contrast, Bansal et al.^33^ reported a significant decrease in DC of resin cement in 4 mm thick leucite-reinforced ceramic or IPS e.max Press compared with 2 or 3 mm plates. Similarly, Oliveira et al.^34^ found that less DC of resin cement was shown under 3.0 mm e.max than under 1.5 mm e.max, and Runnacles et al.^21^ found that less resin cement was polymerized under 2.0 mm than 0.5 mm feldspathic porcelain. These contradictory results about the effects of ceramic thickness on the degree of polymerization of underneath resin cement might be attributed to different experimental conditions such as ceramic specimen thickness, type of resin cement material, the light curing unit, and post-polymerization effects. There have been a number of studies on resin cement post-polymerization during storage after light curing. In previous studies, the DC and hardness of dual-cure resin cement increased as post-irradiation time increased.^24,35^ We measured the DC over the course of time up to 24 h (data not shown), and we detected a slight increase in DC at 24 h in all groups that maintained the between-group differences. However, the increase in DC after light curing was insignificant, we believe, which is because polymerization was induced solely by light cure with no chemical cure.

Although we tried to simulate the clinical surface conditions of ceramic restorations by polishing the top surface and leaving the bottom surface unpolished considering the CAD/CAM fabrication procedure, one limitation of this study is that the bottom was not treated with hydrofluoric acid and silane which might change the light penetration and the DC of resin cements. The influence of surface treatments on the light penetration needs to be investigated in the future. Another limitation of this study is that the DC measurement was performed not in the body temperature but in the room temperature, which could lead a different DC results from the real clinical situation.

Within the limitations of this study, the glass ceramic type and thickness significantly influenced the light transmittance and light spectra and the DC of resin cement underneath ceramics of the same shade. It seems that the choice of a ceramic type should not be based solely on strength because the clinical performance relates not only to the ceramic’s strength but also to the physical properties of the resin cement beneath the ceramic, for which light transmittance is the main influence. Additional studies on other physical resin cement properties such as bond strength, microhardness, and fracture resistance should be conducted to confirm the effect of ceramic type on the clinical performance of ceramic restorations. Moreover, studies are needed to determine the behavior of bonded ceramic restorations in oral environments. Long-term clinical studies will eventually produce clear evidence for checking differing clinical performance by ceramic type.

## Materials and methods

### Fabrication of ceramic plates

We purchased five CAD/CAM ceramic blocks in shade A2: high-translucency and low-translucency types of IPS Empress CAD (Emp_HT and Emp_LT, Ivoclar Vivadent, Schaan, Liechtenstein) for leucite-reinforced glass; high-translucency and low-translucency types of IPS e.max CAD (Emx_HT and Emx_LT, Ivoclar Vivadent) for lithium disilicate glass; and Vita Mark II (Vita Zahnfabrik, Bad Sackingen, Germany) for feldspathic (Table 4). We cut the ceramic blocks into 10 mm squares with final thicknesses of 0.5, 1.0, 2.0, and 4.0 mm using a 3-axis cutting machine (Steptool, Harig, IL, USA). The prepared IPS e.max CAD plates were prepared to have final property. We polished the top surfaces of all plates with 1 µm grit diamond particles with a lapping machine (SPL-15, Okamoto Corp., Santa Fe Springs, CA, USA) in order to simulate the polished surfaces of ceramic restorations. In this way, we fabricated three ceramic plates for each thickness of each ceramic group.

**Table 4:** Ceramic materials and resin cement used in this study

Material (code)		Basic chemical structure (chemical components)	Manufacturer	Lot no.
IPS Empress CAD	HT (Emp_HT)	Leucite-reinforced glass ceramic (SiO_2_, BaO, Al_2_O_3_, CaO, CeO_2_, Na_2_O, K_2_O, B_2_O_3_, TiO_2_)	Ivoclar Vivadent, Schaan, Liechtenstein	P17582
	LT (Emp_LT)			L41778
IPS e.max CAD	HT (Emx_HT)	Lithium disilicate glass ceramic (SiO_2_, Li_2_O, K_2_O, P_2_O_5_, ZrO_2_, ZnO, Al_2_O_3_, MgO)	Ivoclar Vivadent, Schaan, Liechtenstein	P20492
	LT (Emx_LT)			P43213
Vita Mark II (Vita)		Fine-particle feldspathic ceramic (SiO_2_, Al_2_O_3_, Na_2_O, K_2_O, CaO, TiO_2_)	Vita Zahnfabrik, Bad Säckingen, Germany	17510
Variolink N		Base paste: Bis-GMA, UDMA, TEGDMA	Ivoclar Vivadent, Schaan, Liechtenstein	U11257

### Measurement of translucency, light transmittance, and light spectra through the ceramic plates

#### Translucency parameter

We evaluated the translucency of the ceramic plates employing the translucency parameter. We measured each plate’s color with white and black backings using a colorimeter (Minolta Chroma Meter CR-321, Minolta Co., Ltd., Osaka, Japan) in triplicate. For each color measurement, the values were expressed as CIELAB parameters (L*, a*, and b*). L* is lightness, where 100 is completely white and 0 is completely black, and a* and b* are red-green and yellow-blue chromatic coordinates, respectively. A positive a* or b* value represents a red or yellow shade, respectively.^36^ Light source illumination corresponded to average daylight (D65). The colorimeter was calibrated before each measurement period using the white calibrating sample supplied by the manufacturer. The white and black backgrounds used in this study were white (L* = 91.53, a* = 0.87, b* = 3.47) and black (L* = 3.92, a* = 1.50, b* = **−**0.57) ceramic tile.

The translucency parameters of the materials were calculated in the differing thicknesses using the following equation:^30,31^

$${\mathrm{Translucency}}\,{\mathrm{parameter}} = \\ \left\{ {\left( {L^\ast _{\mathrm{W}} - L^\ast _{\mathrm{B}}} \right)^2 + \left( {a^\ast _{\mathrm{W}} - a^\ast _{\mathrm{B}}} \right)^2 + \left( {b^\ast _{\mathrm{W}} - b^\ast _{\mathrm{B}}} \right)^2} \right\}^{1/2},$$

where the subscripts W and B refer to the CIELAB values for each specimen on white backing and black backing, respectively.

#### Light transmittance rate and light spectra through the ceramic plates

We measured the power (mW) of a conventional LED light-curing unit (Bluephase G2, Ivoclar Vivadent) using a photodetector (918D-SL-OD3, Newport Corp., Irvine, CA, USA) attached to an optical power meter (Model 1918-C, Newport Corp.). The light intensity (mW·cm^−2^) was calculated as the ratio of the emitted power (mW) to the area of the light guide tip (cm^2^). The light intensity of the LED light-curing unit was 678 mW·cm^−2^. We then measured the light intensity transmitted through each ceramic plate (0.5, 1.0, 2.0, and 4.0 mm thick) in the five different ceramic materials. The light guide tip was placed in direct contact with the ceramic plates.

The light transmittance rate (%) was calculated as the percentage ratio of the light intensity through a ceramic plate to the light intensity without the plate. When we measured the intensity without the plate, the photodetector was separated from the light guide tip by the corresponding thickness of the ceramic plate. Every measurement was taken 10 s after the light was turned on in order to obtain stable light intensity. We also took triplicate measurements per specimen and calculated the averages.

The light spectra transmitted through each ceramic plate was recorded using a spectrally resolving fiber optic spectrometer (Avaspec-3648, Avantes, Broomfield, CO, USA), and every measurement was taken 10 s after the light was turned on.

### DC of resin cement underneath ceramic plate

We used one ceramic plate of the three plates we prepared for each thickness of each ceramic to measure the DC of resin cement because the differences in values among the three plates in each group were statistically ignorable for the translucency parameter and light transmittance.

The DCs for the resin cements underneath the ceramic plates were examined using Fourier transform infrared spectroscopy (FT-IR) (1600 Series; PerkinElmer, Wellesley, MA, USA). We used only base paste from the base and catalyst of the Variolink N (Ivoclar Vivadent) to evaluate DC solely by light cure. We placed the base paste between two polyester strips, covered each one with a glass slide, and then pressed each firmly against another glass slide base to make a film as thin as possible; the cement film with the two polyester strips was approximately 50 µm thick in all specimens. We fabricated five resin cement films for each ceramic plate, placing each plate over a cement film with the polished surface of the plate facing upward. We placed the light guide tip of the light-curing unit directly onto the polished ceramic surface and light-cured the sample for 40 s. We performed the light curing directly on the resin cement films for the DC control group, which were not influenced by the ceramic plates. Fabrication of resin cement films and measurement of DC by FT-IR was performed in room temperature of dark room to avoid the additional polymerization progress by ambient light. All resin cement films were also kept in aluminum foil-covered petri dish until DC measurement.

Infrared spectra were collected between 1700 cm^−1^ and 1500 cm^−1^ in transmission mode under 4 cm^−1^ resolution and 30 scans. We calculated the DC using the standard baseline method, that is, by the changes in the ratios between the absorbance peaks corresponding to the aliphatic (1637 cm^−1^) and aromatic (1608 cm^−1^) carbon double bonds prior to and after polymerization. We used the absorbance intensities of aromatic C=C as internal references because the intensity does not change during the polymerization reaction. The DC was determined using the following equation:

$${\rm {DC}} \left( {{\% }} \right) = \\ \left[ {1 - \frac{{{\rm{abs}} \left( {{\rm{C}} = {{{\rm{C}}_{\rm{aliphatic}}}}} \right){\rm{/abs}} \left( {{\rm{C}} = {{{\rm{C}}_{{\rm{aromatic}}}}}} \right)\ {\rm{polymer}}}}{{{\rm{abs}} \left( {{\rm{C}} = {{{\rm{C}}_{{\rm{aliphatic}}}}}} \right)/{\rm{abs}} \left( {{\rm{C}} = {{{\rm{C}}_{{\rm{aromatic}}}}}} \right)\ {\rm{monomer}}}}} \right]\times 100,$$

where abs (C=C_aromatic_) is the height of the aromatic C=C bond peak and abs (C=C_aliphatic_) is the height of the aliphatic C=C bond peak for both cured and uncured resin cements.

### Statistical analysis

We conducted two-way analysis of variance to evaluate the differences in the ceramics’ translucency parameters and light transmittance and the DC of the resin cement according to the ceramic type and thickness. We conducted multiple comparisons using the Bonferroni test, and we also ran Pearson’s correlation tests between the translucency parameter and light transmittance rate. For all of the analyses, we used the statistical software SPSS for Windows, v23.0 (IBM Corp., Chicago, IL, USA) with a 95% level of confidence.

## Clinical relevance

Light transmittance differs by the glass ceramic type even in the same shade, which might significantly influence the clinical performance of the resin cements underneath ceramic restorations.
